# Evaluation of VDT-Induced Visual Fatigue by Automatic Detection of Blink Features

**DOI:** 10.3390/s22030916

**Published:** 2022-01-25

**Authors:** Zhijie Yin, Bing Liu, Dongmei Hao, Lin Yang, Yongkang Feng

**Affiliations:** 1 Faculty of Environment and Life, Beijing University of Technology, and Beijing International Science and Technology Cooperation Base for Intelligent Physiological Measurement and Clinical Transformation, Beijing 100124, China; yinzj@emails.bjut.edu.cn (Z.Y.); yanglin@bjut.edu.cn (L.Y.); fengyongkang77@emails.bjut.edu.cn (Y.F.); 2 Ophthalmology Department, The University Hospital of Beijing University of Technology, Beijing 100124, China; liubing1@bjut.edu.cn

**Keywords:** blink feature, incomplete blink, visual display terminal, visual fatigue

## Abstract

This study evaluates the progression of visual fatigue induced by visual display terminal (VDT) using automatically detected blink features. A total of 23 subjects were recruited to participate in a VDT task, during which they were required to watch a 120-min video on a laptop and answer a questionnaire every 30 min. Face video recordings were captured by a camera. The blinking and incomplete blinking images were recognized by automatic detection of the parameters of the eyes. Then, the blink features were extracted including blink number (BN), mean blink interval (Mean_BI), mean blink duration (Mean_BD), group blink number (GBN), mean group blink interval (Mean_GBI), incomplete blink number (IBN), and mean incomplete blink interval (Mean_IBI). The results showed that BN and GBN increased significantly, and that Mean_BI and Mean_GBI decreased significantly over time. Mean_BD and Mean_IBI increased and IBN decreased significantly only in the last 30 min. The blink features automatically detected in this study can be used to evaluate the progression of visual fatigue.

## 1. Introduction

With the development and popularization of the internet, visual display terminals (VDTs), including computers, smartphones, and tablets, etc., have become essential parts of modern life. In particular, during the COVID-19 pandemic and social isolation, people have to work at home and communicate by video conferences. E-learning has become a new form for students. Lack of outdoor recreational activities has resulted in people resorting to VDT for entertainment. Prolonged exposure to a VDT increases the load on the eyes [1] and causes symptoms of visual fatigue, such as blurred vision, dry eyes, burning, headache, eye pain, dizziness, and swelling [2]. Visual fatigue is increasing in prevalence among VDT users [3], producing discomfort for extended periods [4,5,6]. Accordingly, clinicians need to evaluate visual fatigue before providing guidance to minimize the severity of these symptoms [7].

Previous research on measuring visual fatigue can be categorized into subjective and objective methods [8,9]. Subjective ocular symptoms and visual fatigue were evaluated using the ocular surface disease index (OSDI), visual analogue scale (VAS), and computer vision syndrome (CVS) score, and dry eye symptom score [10,11,12]. These methods can be influenced by individual perception, mental states, and daily conditions and cannot be used to continuously measure visual fatigue when a user is viewing a VDT. Thus, the development of objective methods for measuring visual fatigue is now an active area of research.

Visual fatigue induced by viewing 2D or 3D displays has been evaluated objectively by the autonomic nervous response measured by electrocardiogram (ECG) [13], electroencephalogram (EEG) [14], galvanic skin response (GSR), photoplethysmogram (PPG), skin temperature (SKT) [15], and magnetoencephalography (MEG) [16]. However, the accuracy of these signals can be degraded by external stimuli or environmental conditions such as loud noises and air temperature. Further, these studies required inconvenient devices or multiple electrodes to be attached to the participant’s body, which can be uncomfortable and become another factor that contributes to user fatigue.

The parameters from the eyes provide direct evidence for assessing visual fatigue [17,18]. Blink is the regular opening and closing of the eyelids to spread the tear film coated on the frontal part of the cornea across the corneal surface [19]. Healthy people blink about 10 to 15 blinks per minute on average. Some studies indicated that blink rate reduced significantly with the increased ocular symptoms [20,21]. Another study showed the blink rate gradually increased over time, but this trend was not significant [22]. Blink completeness and incompleteness are distinguished based on whether the eyelids touch or not [23] or 75% of corneal coverage by eyelids or not [22]. A greater increase in the incomplete blinks was associated with the prolonged VDT tasks and worsening of the symptom [20,21,22].

Blinks could be detected with the electrooculogram (EOG) signal recorded by skin electrodes placed at opposite sides of the eye [19,24]. However, the attached electrodes may interfere with routine VDT tasks and increase visual fatigue. A glasses-type of infrared light-based eye-tracker has been worn to capture eye images to compare the visual fatigue caused by 2D and 3D displays. The blink rate based on the number of black pixels in the pupil area was calculated to indicate visual fatigue [8,9]. However, this device is inconvenient for those usually wearing glasses.

As a non-contact measurement, a video camera was positioned in front of the participant to capture eyelid movement, and video analysis was subsequently conducted to assess blink features [25]. Mostly, blink was manually recognized and counted from a video recording, which consumed much time and effort for long videos. Machine learning algorithms, such as recurrent neural network (RNN) [26] and support vector machine (SVM) [27], have been proposed to differentiate complete blinks from incomplete blinks. However, a large number of video images and manual annotations were required to train the classifiers. The results were not satisfactory due to the influence of eyelashes and eye size [23].

The conventional image processing methods for recognizing blinks are based on the gradient vector of eye information and local texture information [28]. The pixels of each frame are divided into two groups according to the direction and magnitude of the hybrid gradient vectors. The distance between their centers of gravity is used to determine the blink features [29]. However, this method requires higher image quality and accurate localization of the eye region. The eye aspect ratio between the height and width of the eyes was calculated to detect eye blink and alert the driver about drowsiness; however, the accuracy of eye blink rate detection is not high [30]. Besides, as far as we know, the VDT tasks designed in the previous studies were usually no more than 60 min [23], which could not reflect the progression of visual fatigue.

The present study aims to evaluate the progression of visual fatigue with blink features. We propose an algorithm to identify blink and incomplete blink and extract the blink features at different stages of the VDT task.

In the rest of this paper, Section 2 describes experiment setup, methods of blink detection, incomplete blink detection, blink feature extraction and statistical analysis. Section 3 exhibits the results. Section 4 provides the discussion, and conclusions are drawn in the final Section 5.

## 2. Materials and Methods

Firstly, the experiment was set up to record the face video during a VDT task. Then the eyes were located to extract blinking image frames and recognize complete and incomplete blinks. After that, the blink features were extracted and their changes with visual fatigue were analyzed statistically.

The process of blink features extraction was summarized in Algorithm 1, where n is the total video frames during an experiment.
**Algorithm 1.** Blink feature extraction1. for X = 1; X ≤ n; X++2. Read video image3. Detect face and locate eyes4. Obtain blink frame5. Image enhancement and magnification6. Calculation of the distance between the upper eyelid and eye corner7. Recognize incomplete blink8. Blink features are calculated9. The features are stored in a file10. end for

### 2.1. Experiment Setup

#### 2.1.1. Subject

A total of 23 subjects (12 women, 11 men) aged between 23 and 28 years old participated in this study. They were in good health and had normal or corrected normal vision without eye diseases. They usually spent more than four hours a day staring at VDT screens. The day before the experiment, any drugs, coffee, strong tea, and other beverages that might affect their state were not allowed. At the same time, the subjects ensured that they had a normal diet and enough sleep. The subjects were asked to sign a consent after being informed of the study’s aim, potential benefit, and risk. The study was approved by the Ethics Committee of Science and Technology of Beijing University of Technology and was conducted according to the Declaration of Helsinki of the World Medical Association.

A laptop having a 19-inch display screen (Patriot F907W, Beijing, China) with a resolution of 1440 × 900 and 100% brightness was used to play a documentary video. The documentary video called “The Hunt” produced by the BBC is calm and soothing, however, with drastic changes in hue and brightness. A camera (Spedal MF934H, Guangdong, China) was connected to the laptop via a USB port to record the face video during watching the documentary video. It has a resolution of 1920 × 1080 and a frame rate of 60 frames/s. The camera pointing at the subject’s face was started by the laptop and took the face images continuously at 60 frames/s during the experiment.

#### 2.1.2. Questionnaire for Visual Fatigue Assessment

A questionnaire was designed to evaluate visual fatigue with the score. The visual fatigue questionnaire consists of 5 questions with three options of ‘no’, ‘uncertain’ and ‘yes’, scored 0, 1, and 2, respectively. The higher the total score, the more severe the visual fatigue. The questionnaire shown in Table 1. has been approved by two experienced ophthalmologists.

#### 2.1.3. Experimental Procedure

**Figure 1:**
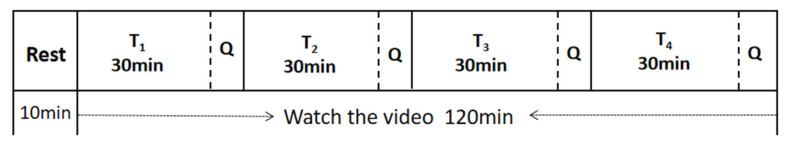
Experimental procedure. Q represents answering the questionnaire orally (about 10 s). T_1_, T_2_, T_3_, and T_4_ are the first, second, third, and fourth 30 min, respectively.

The experiment was carried out at 3 p.m. in a quiet room with constant temperature and ambient light. The subject sat in a chair 50 cm before the display screen facing the camera. Before the start of the experiment, the subjects answered the questionnaire orally and closed their eyes for a 10-min rest. Then, they began to watch the documentary video for 120 min, during which they answered the questionnaire every 30 min. The subject’s face was recorded by the camera during the experiment. The experimental procedure is shown in Figure 1.

### 2.2. Blink Detection

#### 2.2.1. Eyes Location

**Figure 2:**
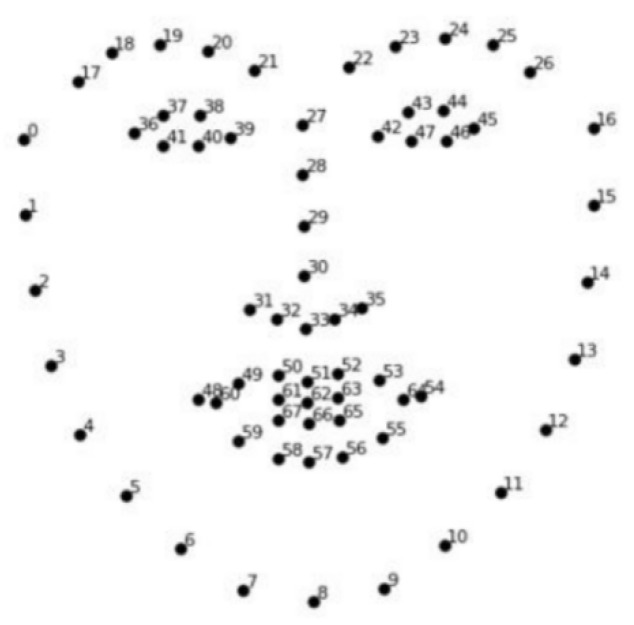
68 key points in a given face.

Firstly, the resolution of the face images was downsampled to 600 × 450 to reduce computation. Then, we applied the advanced facial landmarks to detect the face and locate the eyes [27], in which a set of regression trees was used to estimate the landmark position of the face from a sparse subset of pixel intensities and achieved real-time performance with high-quality prediction. A total of 68 key points of the face were extracted, as shown in Figure 2. The eye area of the target image could be determined with the eye coordinates obtained from the key points on the face. The coordinates of the eyes are from point 36 to 47 and, therefore, the eye images were obtained.

#### 2.2.2. Extraction of Blinking Image Frames

**Figure 3:**
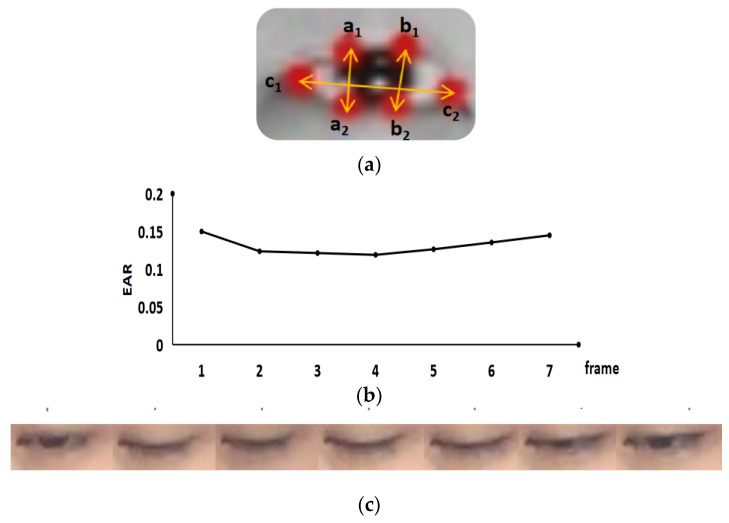
Blink image frame extraction. (**a**) The key points of the eye; (**b**) the change of EAR in a blink; (**c**) the image frames of a blink.

The blinking image frames were captured based on the change of the eye aspect ratio (EAR) [30]. Figure 3a, a_1_, a_2_, b_1_, b_2_, c_1_, c_2_ are the key points on the eye, which were determined by the 68 key points in the face.

EAR was calculated as Equation (1).

(1) $$\mathrm{EAR}=\frac{a_{1}a_{2}+b_{1}b_{2}}{2\times c_{1}c_{2}}$$

where a_1_a_2_ and b_1_b_2_ are the height of the eye, and c_1_c_2_ is the length of the eye.

EAR was calculated for each eye image. It has been observed that EAR is usually larger than 0.2 when the eyes open. Hereby, a blink began when EAR was smaller than 0.2 and ended when EAR was larger than 0.2 again. Figure 3b shows the change of EAR during a blink with seven frames. Figure 3c provides an example of a blink exhibited by seven consecutive images.

### 2.3. Detection of Incomplete Blink

To evaluate whether the incomplete blinks were associated with visual symptoms, this paper detected the incomplete blinks in the following steps.

#### 2.3.1. Extraction of the Eye Contour

**Figure 4:**
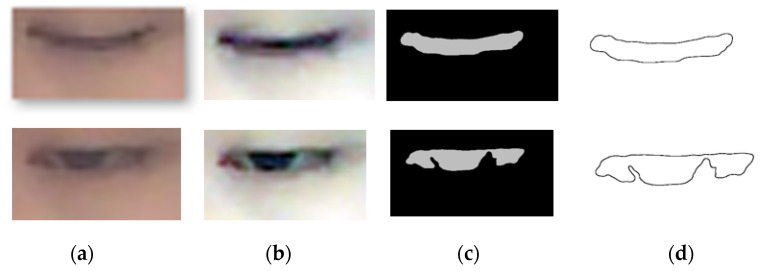
The process of contour extraction. (**a**) Original image; (**b**) SSR image; (**c**) binarized image; (**d**) eye contour.

Firstly, the single scale retinex (SSR) algorithm [31] was applied to improve the brightness, contrast, and sharpness of a greyscale image through a combination of spatial and spectral transformations, which resulted in dynamic range compression and a reduction in the global variance of the image. In our study, the SSR transform scale was set to 300. Then, the eye image was enlarged and enhanced using cubic interpolation with the magnification of 33. After that, the eye image was converted to a grey image and then binarized with the threshold of 139. Finally, the eye boundary was extracted using the adaptive threshold segmentation [32]. Figure 4 shows the contour extraction process.

#### 2.3.2. Calculation of the Distance between the Upper Eyelid and Eye Corner

**Figure 5:**
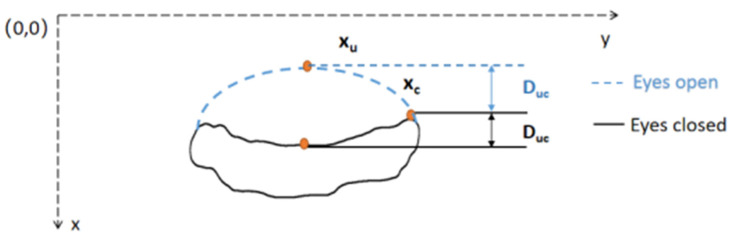
The vertical distance between the midpoint of the upper eyelid and the eye corner. The dashed line stands for eyes open and the solid line for eyes closed.

The vertical distance between the upper eyelid and eye corner expressed by D_uc_ was calculated by the Equation (2), where x_u_ and x_c_ are the *x*-axis coordinates of the upper eyelid midpoint and the eye corner respectively, as shown in Figure 5.

(2) $$D_{\mathrm{uc}}{=x}_{u}\;-\;x_{c}$$

#### 2.3.3. Recognition of Incomplete Blink

**Figure 6:**
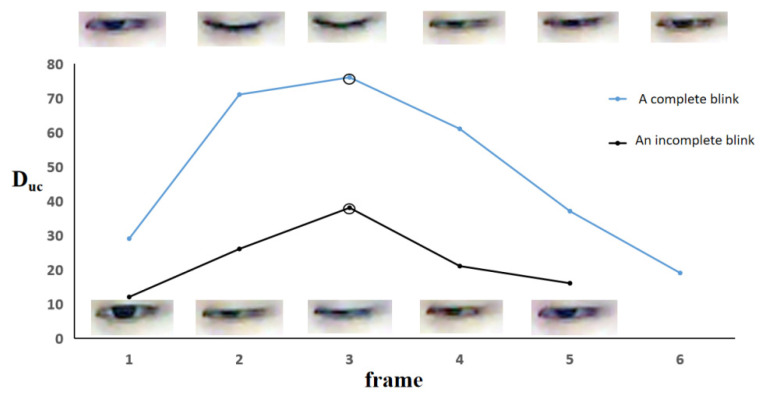
The change of D_uc_ in a blink. The top half corresponds to a complete blink and the bottom half corresponds to an incomplete blinking. ‘°’stands for the maxD_uc_ from a complete and incomplete blink.

We manually labeled the complete blinks and incomplete blinks within the first ten minutes for all subjects, approximately 3000 blinks, including 500 incomplete blinks. D_uc_ was calculated for each frame of blinking images and the maximum D_uc_ was obtained for each blink, denoted as maxD_uc_. The median of maxD_uc_ values of complete blinks within the first ten minutes was acquired as mmD_uc_ for each subject. If the maxD_uc_ value from a blink was less than 75% of mmD_uc_ (the threshold), then this blink was recognized as an incomplete blink, otherwise, it was a complete blink. With this subject-specific threshold, the complete blinks and incomplete blinks were detected for the subsequent 110 min. Figure 6 shows the change of D_uc_ in a blink. The upper part is a complete blink, in which the upper eyelid and lower eyelid touch completely during the eyes closed. The lower part is an incomplete blink, in which the cornea is not completely covered by eyelids during the eyes closed.

### 2.4. Blink Feature Extraction

Blink features were extracted separately from four 30-min videos (phase 1–4), including blink number (BN), mean blink interval (Mean_BI), mean blink duration (Mean_BD), group blink number (GBN), mean group blink interval (Mean_GBI), incomplete blink number (IBN), mean incomplete blink interval (Mean_IBI). These features were defined as follows:

BN is the total number of blinks including complete blinks and incomplete blinks within 30 min.

**Figure 7:**
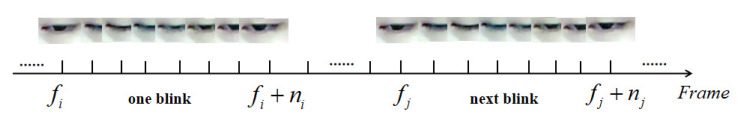
Adjacent blink frame.

Mean_BI is the mean value of the time interval between adjacent two blinks within 30 min. Figure 7 shows the adjacent two blinks, where f_i_ and f_j_ are the start frames, f_i_ + n_i_ and f_j_ + n_j_ are the corresponding end frames. f_j_ − f_i_ is the frame interval between adjacent two blinks.

Mean_BD is the mean blink duration calculated as Equation (3).

(3) $$\mathrm{Mean\_BD}=\frac{1}{\mathrm{fr}\times \mathrm{BN}}\sum_{i}^{\mathrm{BN}}n_{i}$$

where n_i_ is the number of frames recorded in a blink, fr is the frame rate.

GBN is the number of group blinks whose blink interval is less than one second.

Mean_GBI is the mean value of the time interval between the adjacent group blinks.

IBN is the number of incomplete blinks.

Mean_IBI is the mean value of the time interval between the adjacent incomplete blinks.

### 2.5. Statistical Analysis

In this study, the scores of the visual fatigue questionnaire and all blink features were abnormally distributed. The questionnaire score was analyzed by a two-pair Wilcoxon signed-rank test. All blink features from four phases were analyzed by Friedman’s test as a whole, and then pair-wise multiple comparisons were performed. The analysis was performed using the software SPSS 24 (IBM Corp., Armonk, NY, USA). *p* < 0.05 was considered a significant difference. *p* < 0.01 was a very significant difference.

## 3. Results

### 3.1. Analysis of Questionnaire Score

**Figure 8:**
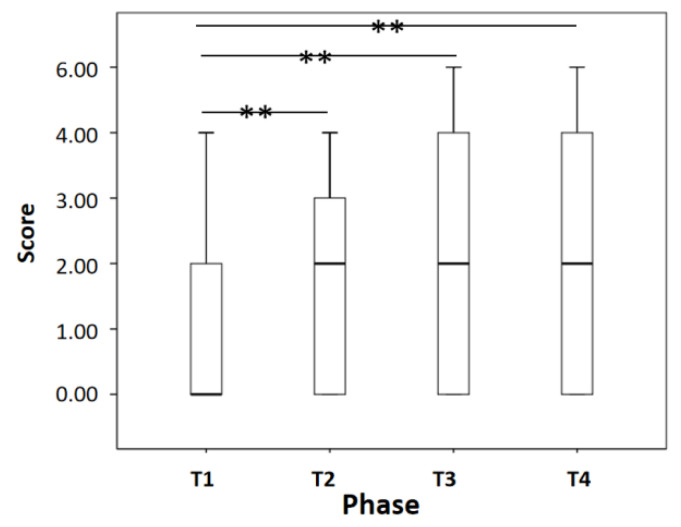
Questionnaire scores. ** *p* < 0.01.

As shown in Figure 8, the questionnaire scores improved very significantly from phase T_1_ to phase T_2_, T_3_, and T_4_ (*p* < 0.01). That is, the symptoms of visual fatigue increased with the time spent watching the video.

### 3.2. Analysis of Blink Features

**Figure 9:**
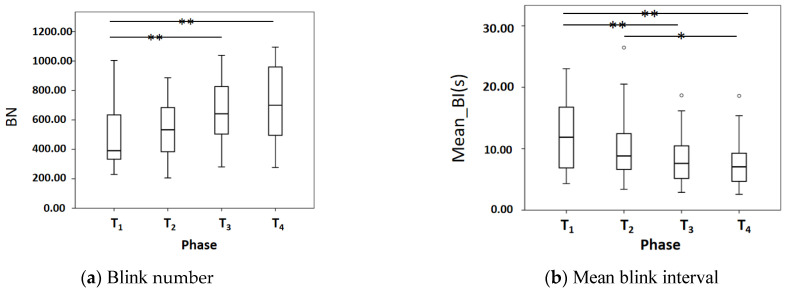

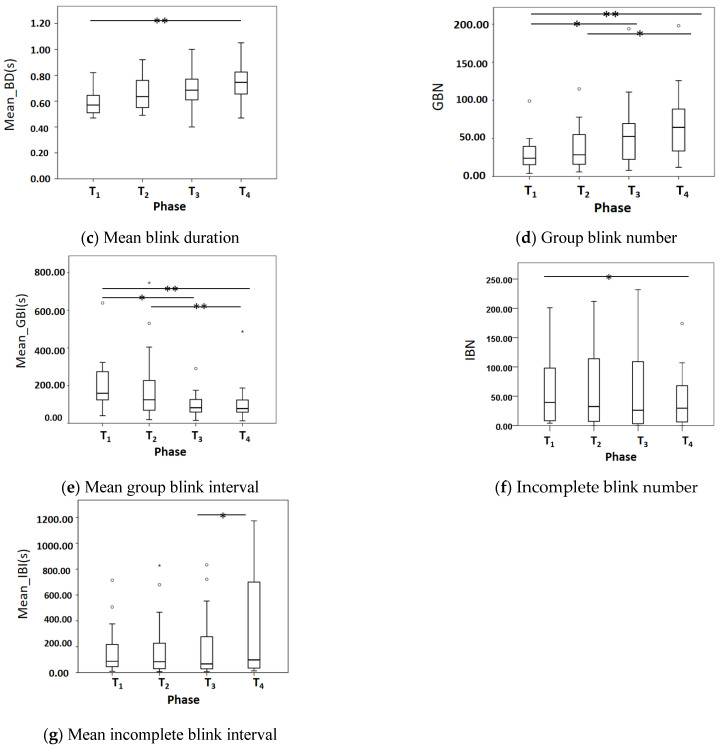
Comparison of blink features. * *p* < 0.05 and ** *p* < 0.01.

Figure 9a shows that BN in the phase of T_3_ and T_4_ are significantly higher than T_1_ (*p* < 0.01). Figure 9b shows that Mean_BI gradually decreases from T_1_ to T_4_, with T_3_ and T_4_ very significantly lower than T_1_ (*p* < 0.01), and T_4_ significantly lower than T_2_ (*p* < 0.05). Figure 9c shows Mean_BD gradually increases from T_1_ to T_4_, with T_4_ very significantly higher than T_1_ (*p* < 0.01). Figure 9d shows GBN gradually increases from T_1_ to T_4_, with T_4_ very significantly higher than T_1_ (*p* < 0.01), T_3_ and T_4_ significantly higher than T_1_ and T_2_ respectively (*p* < 0.05) Figure 9e shows that Mean_GBI in T_4_ is very significantly lower than T_1_ and T_2_ (*p* < 0.01) and T_3_ is significantly lower than the T_1_ (*p* < 0.05). Figure 9f shows that IBN in T_4_ is significantly lower than T_1_ (*p* < 0.05). Figure 9g shows that Mean_IBI in T_4_ is significantly higher than T_3_ (*p* < 0.05).

### 3.3. Summary of the Blink Features at Different Phases

The blink features are summarized in Table 2. Friedman’s test is present as well to indicate the total difference between phase T_1_ and T_4_.

## 4. Discussion

This study designed a long-term VDT task with face video recordings to track the progression of visual fatigue. It did not cause any inconvenience and was more convincing than a short-term task [23]. The blink features extracted from the eye images could reflect the visual fatigue objectively.

A questionnaire was simplified to investigate the subjective feeling without interrupting the VDT task. The increase of the questionnaire score over time was associated with a worsening of visual fatigue.

We proposed the algorithms with the parameters of EAR and D_uc_ to detect blinks, mmD_uc_ and maxD_uc_ to detect incomplete blinks, which eliminated the individual differences with subject-specific parameters. Computations were minimized using the eye’s size and making the algorithm as simple as possible and easy to implement.

In the present study, seven blink features were measured and compared over the 120-min VDT task to indicate the progression of visual fatigue objectively. With the extension of the VDT task, blink number and mean blink duration significantly increased and mean blink interval significantly decreased, which agreed with the previous work. It has been reported that a high blink rate usually occurred at a high degree of visual fatigue [33]. When staring at VDT over a long period, the tear film evaporated excessively, causing the external type of visual fatigue [24]. To relieve the discomfort of visual fatigue, people refreshed the tear film by frequent blinking and prolonging blink duration [34] and, consequently, reducing blink interval. Besides, the increased group blink number and reduced mean group blink interval indicated that the single blink was insufficient to sustain the over evaporated tear film and, therefore, more group blinks participated in the maintenance of tear film and relief of eyestrain.

In respect to incomplete blink, no significant difference was found in both the number and interval among phases 1–3. In phase 4, the incomplete blink number significantly decreased, and the mean interval increased. However, it has been reported the percentage of incomplete blinks increased with visual symptoms during the VDT task [22,25], which is inconsistent with our results. We speculated that the subjects in our study reduced their incomplete blinks and enhanced complete blinks to fight visual fatigue.

Young subjects were recruited in this study, because they usually spend more time on computers or smartphones and, therefore, are susceptible to VDT-induced visual fatigue. For the elders, their visual fatigue may be due to the aging of the eyes. Therefore, this study could be applied to monitor visual fatigue in office workers.

Future work should be directed to improve the image quality and the accuracy of incomplete blinks. Besides, more eye movement parameters could be explored to recognize visual fatigue and even grade visual fatigue. Further, dry eye and mental fatigue will be investigated to disclose their influence on visual fatigue.

## 5. Conclusions

We designed a long-term VDT task and recorded the face video that could capture the progression of visual fatigue without causing any inconvenience to the participants. Based on the ratio of height to length of the eye, a blink could be detected first. Among the blinks, the vertical distance from the midpoint of the upper eyelid to the corner of the eye was calculated to distinguish the incomplete blinks. These automatic blink recognition algorithms have the advantages of low computation and are subject-specific.

We analyzed the blink features statistically and found the blink number, mean blink duration, and group blink number increased, and the mean blink interval and mean group blink interval decreased significantly over time. In terms of incomplete blink, no significant difference was found in the first 90 min; however, the incomplete blink number decreased, and the mean interval increased significantly in the last 30 min.

With the development of visual fatigue, the blink features proposed in this study changed significantly. Therefore, the blink features automatically detected in this study could be used to evaluate the progression of VDT-induced visual fatigue objectively.

## Figures and Tables

**Table 1:** The visual fatigue assessment questionnaire.

Question	Option (Score)		
(a) Dry and burning eyes?	No (0)	Unsure (1)	Yes (2)
(b) Eye pain or foreign body sensation?	No (0)	Unsure (1)	Yes (2)
(c) Blurred vision?	No (0)	Unsure (1)	Yes (2)
(d) Difficulty concentrating?	No (0)	Unsure (1)	Yes (2)
(e) Headache or dizziness?	No (0)	Unsure (1)	Yes (2)

**Table 2:** The median of blink features at different phases.

Feature	Median of Blink Feature				Friedman Test (*p*-Value)
	T_1_	T_2_	T_3_	T_4_	
BN	443	545	664 **	708 **	<0.001
Mean_BI(s)	11.87	9.82	7.61 **	7.05 **^(b^ *^)^	<0.001
Mean_BD	0.57	0.63	0.68	0.75 **	<0.01
GBN	25	38	61 *	71 **^(b^ *^)^	<0.001
Mean_GBI(s)	170.40	126.37	82.17 *	75.06 **^(b^ **^)^	<0.001
IBN	46	33	32	33 *	<0.05
Mean_IBI(s)	87.69	84.03	67.24	98.58 ^(a^ *^)^	<0.05

* *p* < 0.05 and ** *p* < 0.01, comparison with phase 1; ^(a *)^ *p* < 0.05, comparison with phase 3; ^(b *)^ *p* < 0.05 and ^(b **)^ *p* < 0.01, comparison with phase 2.

## Data Availability

The data used to support the findings of this study are included in the article.
